# Preventing occupational injuries in the informal construction industry: a study protocol for the development of a safety education intervention for bricklayers and carpenters in Osun State, Nigeria

**DOI:** 10.3389/fpubh.2024.1464797

**Published:** 2024-10-09

**Authors:** Temitope Olumuyiwa Ojo, Adedeji Ayodeji Onayade, Nisha Naicker

**Affiliations:** ^1^Department of Community Health, Obafemi Awolowo University, Ile-Ife, Osun State, Nigeria; ^2^Division of Occupational Health, School of Public Health, Faculty of Health Sciences, University of the Witwatersrand, Johannesburg, South Africa; ^3^Epidemiology and Surveillance, National Institute for Occupational Health, National Health Laboratory Services, Johannesburg, South Africa

**Keywords:** occupational injuries, Nigerian artisans, safety intervention, construction artisans, carpenters, bricklayers

## Abstract

**Background:**

Occupational injuries are a growing public health problem. Approximately 1,000 workers die daily from occupational injuries globally. Artisans working in the informal sector of the construction industry in many low/middle income countries like Nigeria have a higher injury predisposition. This study will assess the determinants of occupational injuries and design a safety intervention for informal sector artisans in the Nigerian construction industry.

**Methods:**

A sequential mixed-methods design will be employed to study 840 bricklayers and carpenters (420 per artisan group) in Osun State, Nigeria. Quantitative data will be collected first while qualitative data will be collected thereafter. Thirdly, a modified Delphi-technique will be employed to co-design a safety education intervention. For the quantitative study, artisans will be recruited via multi-stage sampling and a semi-structured questionnaire will be administered to obtain information on artisans’ socio-demographics, work-patterns and occupational injuries. A multivariable regression model will be used to determine the association between injury occurrence and independent variables. Twelve to sixteen focus group discussion (FGD) sessions will be conducted for artisans to obtain group perspectives about injuries and preferred safety training topics. From the FGD and quantitative study findings, a list of items for the safety training module will be compiled for the modified-Delphi process. Thereafter, the content validation index (CVI) will be derived and items with CVI of ≥0.80 will be included in the final safety training module.

**Conclusion:**

This paper describes the process required to assess the determinants of occupational injuries among artisans in the informal sector of the construction industry in Nigeria and further proposes the design of a context-relevant safety training intervention. The information from this study will be essential in promoting safe working environments for construction artisans.

## Introduction

Injury at work is an issue of public health importance. An estimated 860,000 persons get ill or injured at work daily (1) and about 1,000 workers on average die daily from occupational injuries globally (2). Some studies in Africa have reported one-year prevalences of occupational injuries among construction workers ranging from 32.4 to 57.9% (3–7). Low-income countries are believed to have a higher prevalence of occupational injuries but they are mostly under-studied and unreported (8). In addition, many low income countries with high burdens of occupational injuries have poor surveillance systems to monitor and control this occupational health problem (9, 10).

The informal sector dominates the economy of many low-income countries and it operates largely without oversight by regulatory bodies (11). In addition, workers in the informal sector are mostly unskilled, poorly trained, casual and daily paid artisans who toil in poor working environments without protective equipment (12). Artisans working in the informal sector of the construction industry have hazardous job exposures that may predispose them to injuries. It is well documented globally that informal sector workers are often harder to study than their formal sector counterparts (13). For instance, in Nigeria, there are many studies on occupational injuries among other workers or professional groups like nurses (14–17), drivers (18–20), factory workers (21, 22), fishermen (23), peasant farmers (24, 25), and police officers (26, 27). However, only a few studies have explored this health problem among informal sector artisans in the construction industry (28, 29).

**Figure 1:**
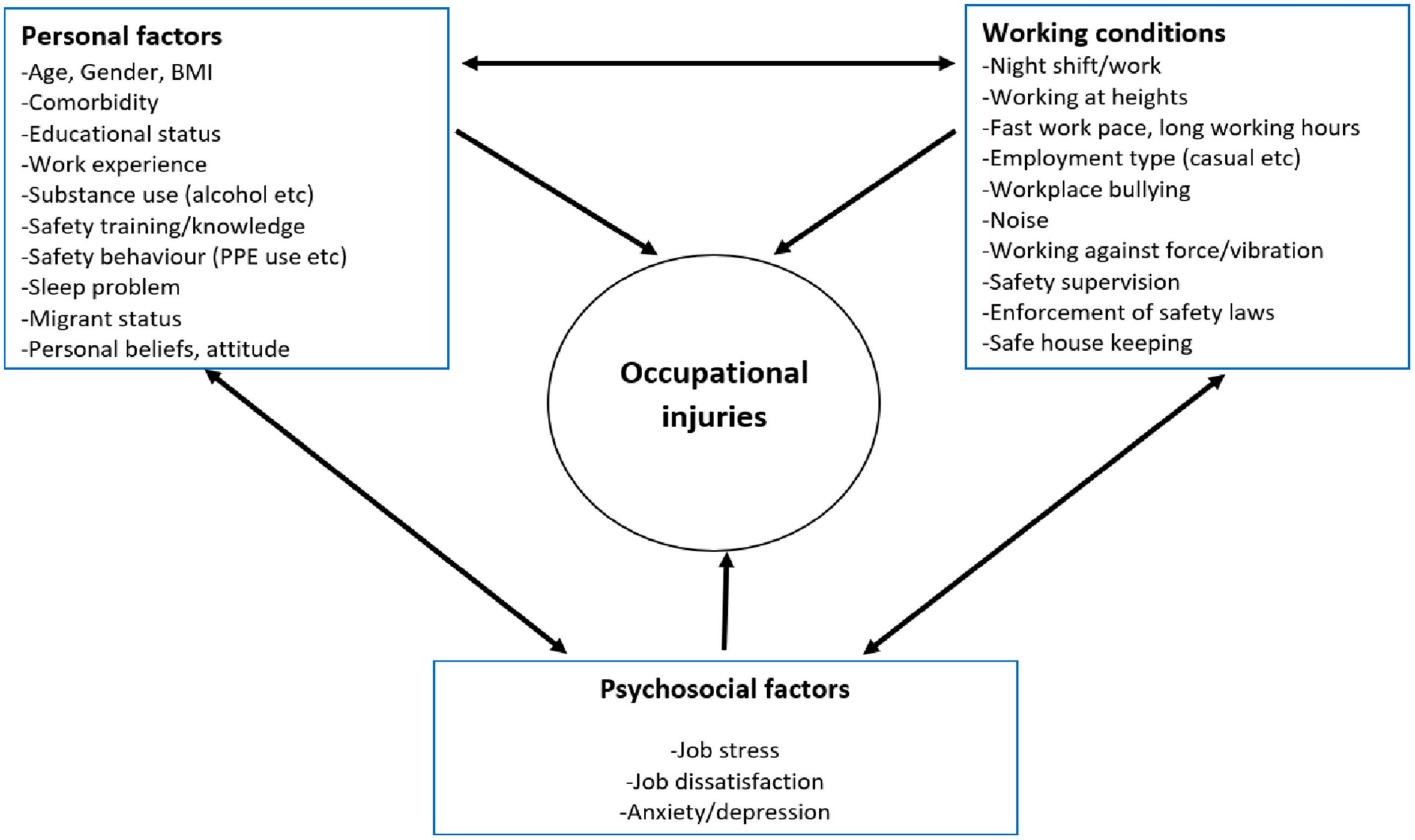
Conceptual framework of factors associated with occupational injuries among construction workers (adapted from the literature review (30–35)).

Previous studies in Nigeria focused on artisans in the formal sector of the construction industry and reported a prevalence of occupational injuries at 36.5% (28). These included artisans (e.g., masons and carpenters) and professional groups (e.g., engineers and architects) employed by large construction firms with better working conditions and regulations. Studies among informal sector artisans are needed because they are major stakeholders in the construction industry, who often work in precarious job settings without safety protection. Working conditions (30, 31), psychosocial and personal factors in the work environment have all been identified as predisposing workers to injury (Figure 1). The conceptual framework aims to explain the interconnectedness of various factors that may predispose vulnerable artisans to injuries. Poor working conditions may influence occupational injuries as shown in past studies. These conditions include lack of health and safety supervision, extended work hours, night work, handling heavy loads, fast work pace and working at heights without safety harnesses (28, 30, 31).

Psychosocial factors in the work environment have been implicated in the growing problem of occupational injuries among the general working population (32, 33). Whether this is true for informal artisans in the Nigerian construction industry remains to be investigated. Past studies have identified job stress, job dissatisfaction and workplace bullying, as important psychosocial factors influencing occupational injuries (5, 6, 34). Other psychosocial factors that have been reported to affect the prevalence of injuries include stress, anxiety and depressive symptoms. These psychosocial factors influence safety behaviors, particularly in relation to self-perceived risk and how individuals respond to potentially hazardous situations (35). Most studies that identified these pertinent psychosocial factors were conducted in high-income countries. Thus, some factors might not be applicable in a low and middle income setting such as Nigeria.

**Figure 2:**
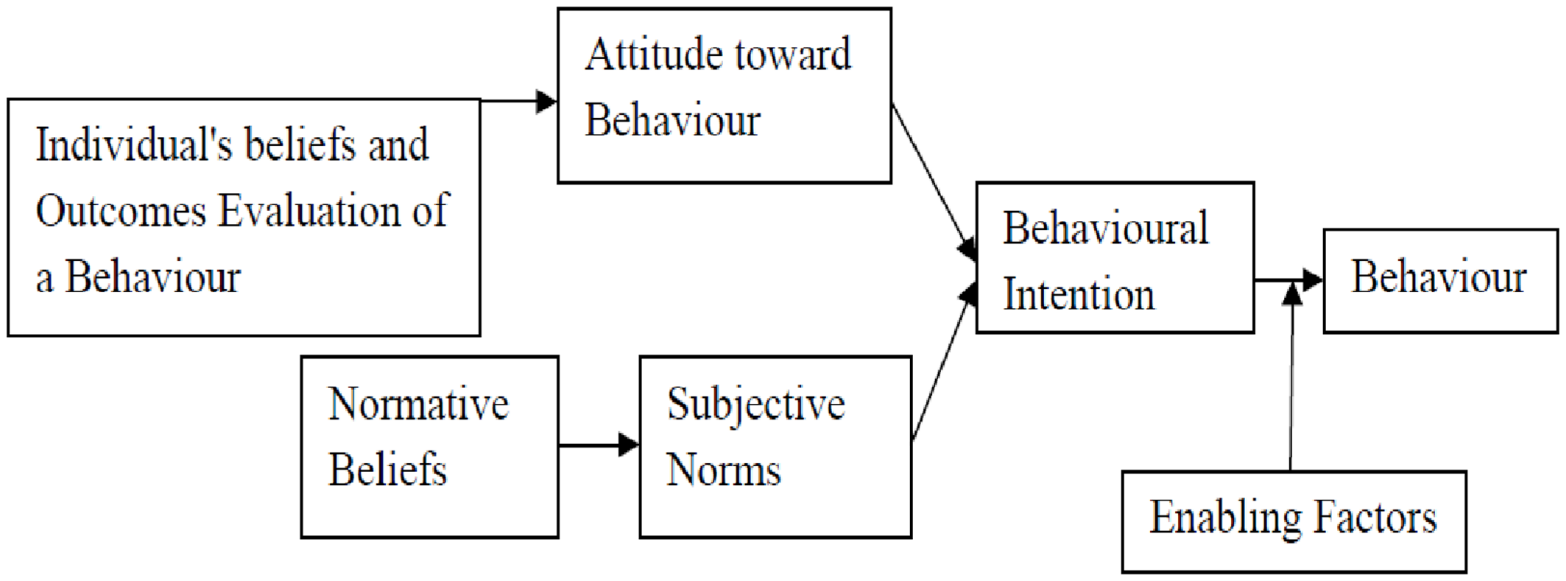
The schematic diagram of the BASNEF model (56).

Furthermore, there remains a paucity of data on the influence of informal artisans’ personal factors on the prevalence of workplace injuries. Personal factors may present a window of opportunity for behavior change communication targeted at each worker. It has been shown in past studies outside of Nigeria that young age, male sex, migrant status, poor safety training, fewer years of experience, lack of formal education and sleep problems, are all associated with a higher likelihood of injuries in construction workers (7, 36, 37). Other personal factors associated with injuries are substance use (e.g., alcohol and cannabis), poor and inconsistent use of PPE and the presence of comorbidities (36). In addition, past studies did not design safety interventions aimed at improving the knowledge and behavioral intentions of artisans in the informal sector of the construction industry. This study hopes to address this gap by using a BASNEF (belief, attitude, subjective norms, and enabling factors) model-based safety training intervention (Figure 2). The model highlights the importance of some key constructs such as personal beliefs, attitudes as well norms and enabling factors in predisposing to a particular behavior or outcome. The main advantage of this model is that it emphasizes a wider range of factors at the individual level that can be targeted for positive behavior change. Some factors such as personal knowledge, beliefs and safety behavior among others in the conceptual framework in Figure 1 will be incorporated into the BASNEF model while designing the safety training intervention. This will help identify relevant and modifiable factors while ensuring that the intervention is well grounded in theory and more likely to be effective, especially in the study context.

The BASNEF model is an effective model in health promotion and education especially in worker’s safety training (38) but the model’s relevance is yet to be examined among Nigerian construction artisans. The BASNEF model has been used to design behavioral change interventions and determine factors that may influence an individual’s behavior or behavioral intentions (38). The BASNEF model-based interventions have been used to improve safety practices among workers, such as tile industry workers (39), carbon block factory workers (38) and sawmill workers (40) in some low and middle-income countries.

The Nigerian construction industry is dominated by artisans, particularly in the informal sector, where craftsmen such as bricklayers, plumbers, carpenters, tilers, iron benders, and painters play a significant role. These artisans have received relatively scant attention in the literature. Existing studies indicate that among construction artisans, bricklayers and carpenters are especially vulnerable to injury and thus require further research (41–43). Therefore, this study will focus on bricklayers and carpenters as a starting point, with plans to include other artisans in future research.

Furthermore, this study will focus on important gaps identified and assess factors influencing occupational injuries and the design of a context-relevant safety training intervention on safety knowledge and practice. The study findings will inform occupational health policies for informal sector employees in the Nigerian construction industry and provide a basis for advocating and planning basic occupational health services for these vulnerable workers.

## Study objectives

The study aims to identify the factors associated with occupational injuries and design a safety intervention to improve knowledge and safety practices of informal sector artisans in the construction industry in Osun State, Nigeria.

The specific objectives are:

1. Determine and compare the prevalence and pattern (types, severity and mechanism) of occupational injuries among construction artisans (bricklayers and carpenters) in Osun State, Nigeria.
2. Identify the factors (psychosocial, personal, and working conditions) associated with occupational injuries among bricklayers and carpenters in Osun State, Nigeria.
3. Explore and compare perceptions about occupational injury and measures to prevent occupational injuries among bricklayers and carpenters in Osun State, Nigeria.
4. Co-design and validate a safety education program aimed at improving occupational safety knowledge and injury prevention practices among construction industry artisans.

## Methods/design

### Study design

The design is a sequential mixed-methods design. Quantitative data will be collected first while qualitative data will be collected thereafter to provide contextual insights to the findings from the quantitative survey (sequential explanatory). The study shall be conducted in three phases as follows; firstly, the quantitative aspect shall entail a cross-sectional study, while the second phase shall comprise the collection of qualitative data using focus group discussions (FGDs). The qualitative and quantitative data will both be triangulated to answer the study objectives. Thirdly, a modified Delphi-technique will be employed to co-design a safety education program. The study is expected to be conducted between June 2024 and April 2025.

### Study location

The study will be conducted in Osun State, one of the 36 states in Nigeria. Osun State has a population of 5.6 million based on projected estimates of the 2006 national census (44). There are three senatorial districts and 30 local government areas (LGA) in the state. The majority of the inhabitants are traders, artisans, farmers and civil servants. Many informal artisans are engaged in vocations across various locations in the state with most of them working individually or in small groups across shops or different work sites.

These artisans are organized under a self-run association which exists at multiple levels (from zones to LGA, senatorial district and then state level). Information obtained from the leadership of the artisans’ associations revealed that there are about 16,698 registered master carpenters and 19,700 registered master bricklayers in the study location as of April 2024.

## Quantitative data collection

### Study population

Bricklayers and carpenters working in the informal sector of the construction industry will comprise the study population for the first study component. Bricklayers typically lay stones, blocks, and bricks in the construction of building walls and civil works while carpenters are workers who build or repair wooden structures or their structural parts. Bricklayers and carpenters are among the vocations with the highest burden of occupational injuries among informal construction artisans (41, 42), hence this comparison study.

#### Inclusion criteria

Study participants must have worked for at least 1 year in the vocation, be at least 18 years old and provide consent. They must be registered members of an artisan group. Special considerations will be made for persons with physical disability for instance persons with hearing impairment will be encouraged to participate using sign language interpreters or alternative communication methods at no cost to ensure equitable access to the study.

#### Exclusion criteria

Bricklayers and carpenters who are unwilling to participate and those with acute severe illnesses who are unable to respond to interviews.

## Sample size and sampling

### Quantitative study sample size calculation

The formula for estimating sample size for the comparison of proportions across two groups was used in determining the sample size for this study. *N*/per group = (*Z*_α_ + *Z*_ß_)^2^ × [(*P*_1_*Q*_1_) + (*P*_0_*Q*_0_)]/(*P*_1_-*P*_0_)^2^.

Using a *Z*_α_ at 95% confidence limit and *Z*_β_ at 80% confidence limit, P_1_ = Proportion of carpenters who had an injury at work from a past study = 38.5% (28) Proportion of bricklayers who had an injury at work from a past study = 28.3% (28). After adjusting for 10% refusal (non-response) and another 10% for potential confounder (age). A sample size of 420 per group was derived. Thus, a total of 840 artisans shall be recruited into the study.

### Sampling technique for quantitative data collection

Eligible Master artisans shall be recruited over a three-month period via a multi-stage sampling technique.

Stage 1: one out of the available three senatorial districts shall be selected in Osun State using balloting (a form of simple random sampling).

Stage 2: two local government areas LGAs shall be selected out of 10 LGAs in the selected senatorial district using balloting (each senatorial district has 10 LGAs).

Stage 3: five artisan zonal groups shall be selected in each selected LGA using a simple random sampling technique (balloting). The number of zonal groups varies per LGA, but each LGA has an average of 10 zones.

Stage 4: with the established relationship with the executives of the artisan’s group, the list of registered members shall be obtained and 42 members in each selected artisan zonal group shall be recruited through simple random sampling (using a random number table). The list of registered members shall be the sampling frame for the selection process. This sampling procedure shall be conducted for both artisan groups (bricklayers and carpenters) in this study.

## Data collection tools

### Recruitment and training of research assistants

Eight research assistants will be recruited and trained for data collection. They will be persons with a background in health sciences who are nurses/health care workers and with sufficient experience in studies of this nature. They should be fluent in both the native and English languages, and they should be very familiar with the terrain of the study locations. The meaning of every item on the instrument will be properly explained to the research assistants. Training methods will include training sessions, role plays, questions and answers and field practice. Research assistants will be closely supervised by the first author to ensure data quality. Daily uploads will be reviewed for completeness and accuracy. Daily briefings and reviews will enhance data quality through open communication. The first author will offer technical support during data collection, ensuring accuracy and consistency.

### Quantitative questionnaire

The quantitative data will be collected using a semi-structured questionnaire. The questionnaires will be administered with REDCap (45) on tablets using an interviewer method. The questionnaire will be translated into Yoruba, the native language of the study area and back-translated to English by linguistic experts. The questionnaire will be used to obtain information on artisans’ socio-demographics, work patterns, work experience, knowledge and use of personal protective equipment. In addition, information will be obtained about occupational injuries (types, pattern, severity, place and cost of care). Information on safety knowledge and practices, use of PPE, attitude, enabling factors and subjective norms will also be obtained.

Information on the possible factors (personal, psychosocial, and working conditions) influencing occupational injuries will be ascertained. The questions are mostly close-ended, offering different response options to capture the respondents’ answers.

Also, an assessment of sleep status will be conducted using the Jenkins sleep scale (46) and job stress will be assessed using the workplace stress survey scale (47). These scales have been validated and used in Nigeria and similar countries.

### Clinical examinations

Clinical examinations such as visual acuity will be conducted by trained research assistants who are nurses/health care workers. These tests will be conducted as screening tests for study participants at no cost and it is meant to assess the visual status of study participants as this is an important factor in occupational injury occurrence. Other examinations will include height, weight and blood pressure checks to serve as motivation for study participants to participate in the study. As these participants do not access healthcare facilities regularly for health assessments, therefore, this study presents a unique opportunity to avail them of some basic health checks (48, 49). The basic health checks will be conducted at a private corner in their association meeting venue. Persons with abnormalities on clinical examination will be referred as appropriate with the aid of printed referral forms.

### Pre-testing of the quantitative study tool

#### Pre-test

The study instrument will be pre-tested among 30 artisans each across both artisan groups (60 in all) in a local government area not included in the study and this corresponds to about 7% of the study sample size. The pre-test will adjudge respondent’s acceptability of the questions and ease of responding to them. Following the pre-test, ambiguous questions and those that elicit inappropriate responses will be re-structured and re-assessed. The overall reliability of the questionnaire will be assessed using Cronbach’s alpha.

### Qualitative data collection

The FGDs will be conducted to obtain group perspectives about injuries and to harvest opinions on safety training topics and preferred training approach(es). The group dynamics during FGDs may provide more social context and help generate broader ideas needed to design an effective safety training intervention.

### Study population

Bricklayers and carpenters working in the informal sector of the construction industry in Osun State will comprise the study population. The lived experiences of artisans who had major injuries (requiring time off work or needing professional health care) (49) in a recent time (less than 3 months) may be different from those without recent injuries. Therefore, separate FGDs will be held for these two groups to capture their perspectives.

### Sampling technique for qualitative data collection

The respondents/discussants for the qualitative data collection will be artisans who will be purposively selected, based on pre-determined criteria. The criteria will include those who sustained major injuries at work in the last 3 months and those who have not had major injuries in the last 3 months.

The list of registered artisans used for the quantitative study will be reviewed and those who were not selected for the quantitative study will be interviewed and screened to know if they have had major injuries in the last 3 months or not. Serial recruitment will be done until 6–8 eligible discussants are selected for each FGD. Four to six FGD sessions will be held in each of the selected LGAs used for the quantitative study. A total of 12–16 sessions of FGD will be conducted. Separate FGD sessions will be held for bricklayers and carpenters (those who have had major injuries and those who have not had in the last 3 months). Data saturation will be deemed to be achieved when no new themes arise and existing ones are well-developed (50).

### Qualitative study instrument

The study instrument is a focus group discussion guide which was drafted based on the review of literature. Each FGD will be conducted by a moderator and a note-taker and recorded using a tape recorder. The first author who is a male PhD candidate will moderate all FGDs. In addition, the lead author has extensive training in qualitative studies. The FGDs will be conducted in the local language (Yoruba) and each session should last about 1 h. FGDs shall take place in rented halls not far from their association meeting venues and participants shall receive a token for their transport.

### Pretesting of the qualitative study instrument

Two FGDs (which corresponds to 17% of the number of FGDs proposed for the study) will be conducted to pretest the qualitative study instrument in one local government area not included in the study. The feedback from the pretest will be used to improve the study instrument as regards appropriateness and ability to probe and stimulate discussions around ideas related to the study objectives.

### Safety training intervention design using a modified Delphi process

The ‘safety training intervention’ development process entails a modified Delphi process (51, 52) and consists of an expert review of items derived from the FGD findings and insights from the quantitative study. An initial meeting will be held with the heads of the artisans’ association to prioritize and rank the different items identified. Subsequently, the list of identified training priorities will be harmonized and developed into a training format for experts to assess the content and quality of the training content. The expert reviews shall be done in two to three rounds until a consensus is attained as regards the contents of the training module.

### Target population and sampling for the modified-Delphi process

The target population for the modified Delphi process comprises selected experts in Occupational Medicine and Public Health in Nigeria. The experts shall have postgraduate medical education in Public Health and be currently employed as an academic or professional in occupational health practice. In addition, these experts must be familiar with the informal sector of the construction industry in Nigeria. This will ultimately enhance the reliability, relevance, and practicality of the data collected through the Delphi method.

The register of all registered members of the Association of Public Health Physicians of Nigeria (APHPN) will be obtained from the executives. All the registered APHPN members that belong to the caucus/subgroup on Occupational and Environmental Health will be contacted via e-mail.

### Study tool for the modified-Delphi process

A tool for the modified-Delphi process will be used to rank the suggested topics. The suggested topics gleaned from quantitative results and FGD findings will be listed, and the subject matter experts will rank and score the appropriateness while also stating suggestions on how to improve the topics. The data collection for the modified-Delphi process will be conducted in about two or three rounds until a concise training module is produced. The data collection will be conducted online via REDCap.

**Figure 3:**
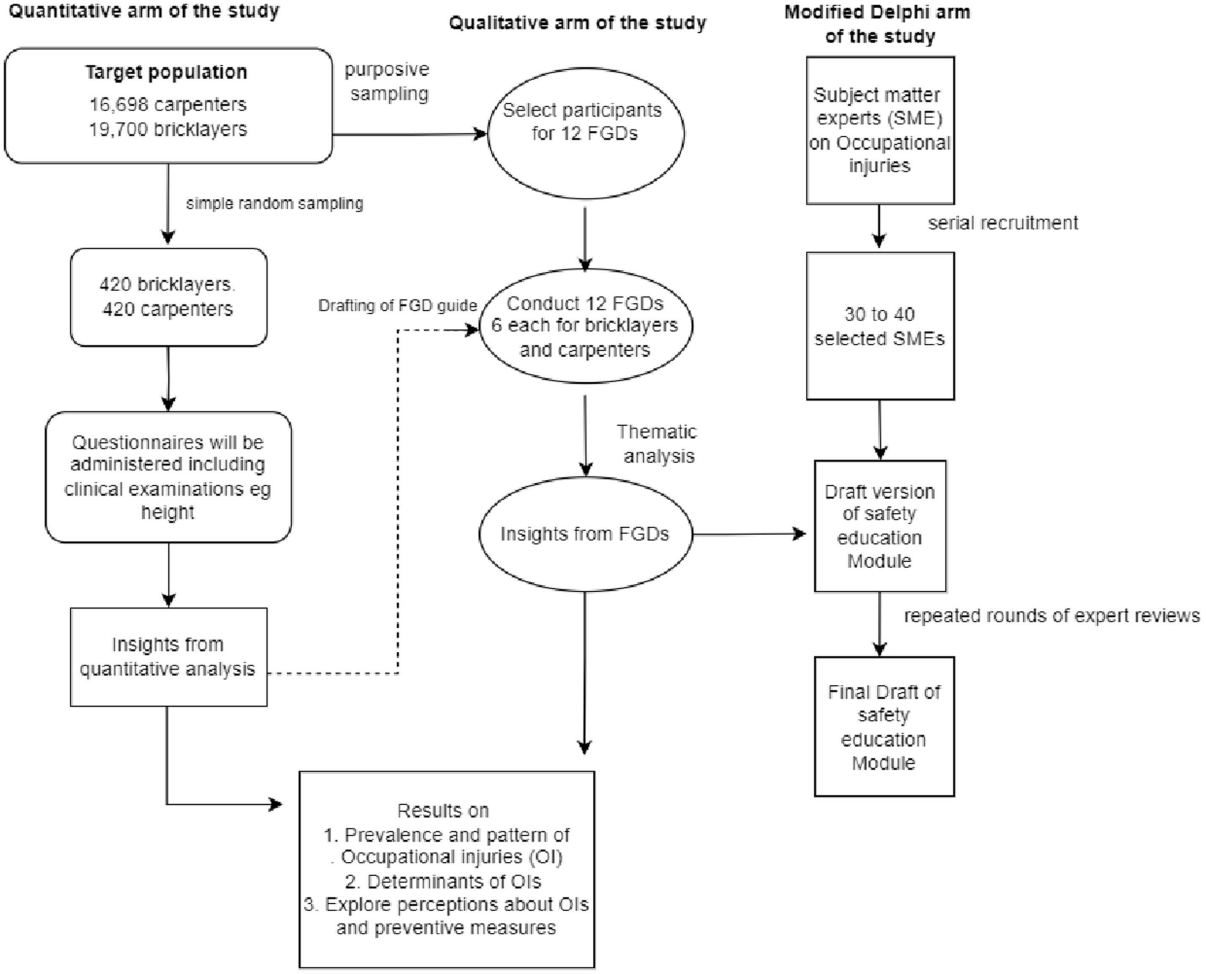
A schema showing the different aspects of the proposed study.

The study tool will be piloted among five selected subject matter experts. The link to the study will be sent to their email address and the responses and feedback on how to improve the data collection tool will be incorporated. The details of the different study arms and how they interlink are shown in Figure 3.

### Data management and analyses

#### Data management

For quantitative data, there shall be a daily upload of completed questionnaires to the REDCap web-based server for real-time monitoring. At the end of quantitative data collection, the data shall be downloaded from the REDCap server in CSV/Excel format for data cleaning and converted to a Stata file for data analysis. For the qualitative data, all recordings shall be downloaded from recording devices, translated from Yoruba and transcribed into English language. Proofreading of all transcripts shall be conducted to correct typographical and other errors in transcription. The transcripts will then be coded for qualitative analysis.

#### Objective 1: (quantitative analysis)

Data analysis will be conducted using Stata version 16.1 (Stata Corporation, United States). The demographic characteristics of artisans (e.g., age and marital status etc.) will be summarized using frequencies and proportions. Results will be presented using charts and tables. Various measures of central tendencies and dispersions will also be used to summarize continuous variables. Comparisons of prevalence, pattern and mechanisms of injury will be conducted using the chi-square test of statistics.

#### Objective 2: (quantitative analysis)

Pearson’s chi-square test will be used to determine the association between categorical variables (e.g., presence or absence of occupational injury and age group). The independent samples t-test will be used to assess differences in means of quantitative variables across artisan groups. Multivariate logistics regression analysis will be conducted to identify factors associated with occupational injuries. The explanatory variables for the regression model shall comprise the sociodemographic variables (age, years of work, educational status, income, migrant status, etc.) and the different workplace factors (workplace conditions, personal and psychosocial factors) while the outcome variable is the occurrence of occupational injury. Variables shall be loaded on the model if they are significant at *p*-values of 0.2 or less. The goodness of fit test of the model shall be performed and interaction between variables shall be assessed. A stratified analysis shall be conducted for the different artisan groups in the study. For all statistical analyses, *p* < 0.05 will be taken as statistically significant.

#### Objective 3: (qualitative analysis)

The English transcripts will be reviewed and codes will be developed to represent words, phrases, clauses or sentences with similar meanings. Deductive coding will be done initially based on the major themes from the FGD guides. However, as more themes emerge from the data, a new set of codes will be generated codes (inductive). The definitions of the codes shall be aggregated in a codebook. Double coding will be done for about 20% of transcripts to increase the reliability of the coding process. Coding and analysis shall be conducted using NVivo version 12.1 software (QSR International Pty Ltd., Australia). The qualitative findings shall be presented using narrations and direct quotes from artisans that best represent the themes identified from the analysis. Some anticipated themes will include the attitudes, norms and beliefs surrounding occupational injuries (OIs) among participants Also the patterns, trends and underlying factors contributing to OIs and lastly practical recommendations for developing effective injury prevention strategies tailored to the study population. Lastly, the theme around suggested training topics and some pertinent findings from the quantitative results will be synthesized to generate a draft list of topics for the training intervention.

#### Objective 4: (quantitative analysis)

The validity of module contents for the safety training will be conducted using the content validation index (CVI) (53). Subject matter experts will examine each training item and rank the contents on a scale (1 for “not relevant”; 2 for “major revision needed”; 3 for “relevant but needs minor revision” and 4 for “very relevant”). The CVI shall be computed by dividing the number of experts that adjudged a content as relevant (that is, ranked 3 or 4) over the total number of experts. Based on the CVI, the proportion of agreement on the relevance of each content will be expressed between zero and one. For this study, only items with a CVI of 0.80 or higher will be included in the final training module, while a CVI of 0.70–0.79 indicates that some revision is required; and a CVI of less than 0.70 indicates that the item should be dropped (54).

### Statistical analyses

During data analysis, the integration of the qualitative and quantitative data will be done to allow each set of findings to deepen, enrich, and complement the other, especially for objectives two and three. This form of concurrent triangulation approach will use qualitative data to explain the findings from the quantitative data, and vice versa.

### Dissemination of information

The artisans will be provided general feedback on the study findings and safety practices that need to be improved upon. The study findings will be presented during seminars at the School of Public Health, University of the Witwatersrand. The Osun State Ministry of Trades and Investment will receive policy briefs on ways to improve safety practices among construction artisans. Pertinent findings from the study will also be presented at scholarly meetings and scientific conferences, while some findings will be published in peer-reviewed journals.

### Ethical considerations

Ethical approval to conduct the study was obtained from the Human Research Ethics Committee (Medical) of the University of Witwatersrand, South Africa (M221066) and the Osun State Health Research and Ethics Committee in Osun State, Nigeria (OSHREC/PRS/569 T/303). In addition, the study will be conducted in line with the ethical standards as laid down in the Declaration of Helsinki and its later amendments or comparable ethical standards (55). Permission to carry out the survey will be obtained from the head of the artisans’ association in the selected LGAs. Written informed consent will be obtained from all adult persons before participating in the survey after an adequate explanation of the purpose of the study and the study’s risks and benefits.

Participation will be voluntary and confidentiality as well as data security will be ensured. Respondents will be informed of their rights to decline or withdraw at any time without penalties. Data will be stored on a computer that is password-protected and only accessible to the investigator. The clinical impression obtained from available information after survey assessments will be communicated to individual respondents. All participants with clinical conditions detected on screening or those with other health complaints will be subsequently referred after due counseling, to an appropriate formal healthcare provider of their choice with the aid of a study referral form.

## Discussion

This paper describes the process required to assess the determinants of occupational injuries among artisans in the informal sector of the construction industry of a typical Low/middle-income country. The paper further proposes a safety training intervention which is grounded in theory.

This research undertakes an assessment of workplace injury burdens in the informal construction sector in a typical low- or middle-income country while considering the intricate web of factors contributing to occupational injuries among artisans. Besides the identification of risks, the development of a theory-driven safety training intervention is being proposed to address the unique vulnerabilities of this often-overlooked demographic. By elucidating the nuanced challenges faced by artisans, this study may provide information needed to catalyze positive change within the sector. By bridging the gap between research and practice, this research seeks to contribute meaningfully to the advancement of occupational safety and health in informal construction settings, ultimately enhancing the holistic well-being and socio-economic prospects of artisans in these contexts.

The proper understanding of factors associated with occupational injuries among construction artisans is important as it will potentiate further research and evidence-informed policy change.

This study has some limitations; for instance, the study will be conducted in Osun State, Nigeria, hence study findings may not be representative of the entire artisans in the nation. In addition, two artisan groups (bricklayers and carpenters) will be recruited into the study. Hence, study findings may not be generalizable to the other types of artisans who also work in the construction industry (e.g., Iron benders, manual laborers, Tilers, Plumbers, Electricians, etc.). In addition, due to the cross-sectional study design, causality cannot be established since exposure and outcome will be assessed at the same time. However, despite these limitations, the study will provide reliable estimates of the burden of occupational injuries and perhaps this study may be the first to design a validated safety education intervention for construction artisans in Nigeria.

## Conclusion

This study’s insights will empower stakeholders to design targeted interventions that not only foster safe working environments and promote decent work for artisans but also stimulate economic growth. For instance, findings could inform the development of culturally relevant safety training programs, advocate for improved access to personal protective equipment, and guide regulatory agencies in strengthening enforcement mechanisms. Equipping artisans’ associations with tailored recommendations for improved safety practices and regulatory bodies with evidence-based strategies may facilitate an informal construction sector that prioritizes both the well-being and economic prosperity of its workforce. Consequently, this may contribute to broader economic development and improved livelihoods for artisans in resource limited settings.
